# Study of genetic variability in *Vitis vinifera* L. germplasm by high-throughput Vitis18kSNP array: the case of Georgian genetic resources

**DOI:** 10.1186/s12870-015-0510-9

**Published:** 2015-06-23

**Authors:** Gabriella De Lorenzis, Ramaz Chipashvili, Osvaldo Failla, David Maghradze

**Affiliations:** Dipartimento di Scienze Agrarie ed Ambientali, Università degli Studi di Milano, Milan, Italy; Institute of Viticulture and Oenology, Agricultural University of Georgia, Tbilisi, Georgia; National Wine Agency of Georgia, Tbilisi, Georgia

**Keywords:** Domestication, Molecular markers, SNP, *V. vinifera* subsp. *sativa*, *V. vinifera* subsp. *sylvestris*

## Abstract

**Background:**

Georgia, in the Caucasian region, is considered the first domestication centre of grapevine. This country is characterized by high morphological variability of cultivated (*Vitis vinifera* L. subsp. *sativa* (DC.) Hegi) and wild (*Vitis vinifera* L. subsp. *sylvestris* (Gmel.) Hegi) compartments. The main objective of this study was to investigate the level of genetic diversity obtained by the novel custom Vitis18kSNP array, in order to analyse 71 grapevine accessions representative of wild and cultivated Georgian germplasms.

**Results:**

The number of loci successfully amplified was 15,317 out of 18,775 SNP and 79 % of loci resulted polymorphic. Sixty-eight unique profiles were identified, 42 for the *sativa* and 26 for the *sylvestris* compartment. Cluster analysis highlighted two main groups, one for cultivars and another for wild individuals, while a genetic structure according to accession taxonomic status and cultivar geographical origin was revealed by multivariate analysis, differentiating clearly the genotypes into 3 main groups, two groups including cultivars and one for wild individuals, even though a considerable overlapping area was observed.

**Conclusions:**

Pattern of genetic diversity structure presented an additional proof that grapevine domestication events took place in the Caucasian region contributing to the crop evolution. Our results demonstrated a moderate differentiation between *sativa* and *sylvestris* compartments, even though a connection between several samples of both subspecies may be assumed for the occurrence of cross hybridization events among native wild populations and the cultivated accessions. Nevertheless, first degree relationships have not been discovered between wild and cultivated individuals.

**Electronic supplementary material:**

The online version of this article (doi:10.1186/s12870-015-0510-9) contains supplementary material, which is available to authorized users.

## Background

Grapevine (*Vitis vinifera* L.) is one of the most widely cultivated species of agricultural interest [1], spread from Central Asia to the Mediterranean Basin [2]. Two subspecies, *V. vinifera* L. subsp. *sylvestris* (Gmel.) Hegi and *V. vinifera* L. subsp. *sativa* (DC.) Hegi, are considered to co-exist. The first one represented by wild populations and the second one represented by cultivated varieties obtained from wild individuals through a domestication process [3]. The two subspecies show differences in several phenotypic traits, one of the most distinctive traits is the flower sex, dioecious for wild grapes and hermaphroditic, or, to a lesser extent, female, for cultivated grapes [4].

The domestication of wild grapes started in the Neolithic Age, about 8,000 years ago, as a result of a long and gradual process closely linked to winemaking [5, 6]. Archaeological remains and proto-historical sources suggest the Near East area, comprising the South Caucasus, Oriental Anatolia, Syria and the area around Northern Mesopotamia, as the first centre of domestication [6, 7]. From the primary domestication areas, the grapevine spread to neighbouring regions and followed different pathways and successive waves firstly towards Mesopotamia, East Mediterranean Basin, North Africa, Southern Balkans and Aegean Region; secondly towards Sicily, Southern Italy, France and Spain; and finally towards Central Europe, mainly through the main trade routes of Rhine, Rhone and Danube rivers [6]. In agreement with these general dispersal pathways, many studies of grapevine genetic diversity supported the hypothesis of secondary domestication centres in the Mediterranean area, considering the crucial role of the Near East in grapevine domestication, and the introgression processes, from wild compartment of the secondary centres of domestication, in the cultivated germplasm, as complementary sources of genetic diversity in the domesticated gene pool [8-12].

A decisive contribution to interpret the molecular diversity of *V. vinifera* and its putative geographic origin was given by the analysis of two large grapevine collections [10, 13]. The first one repository, the grape germplasm collection of US Department of Agriculture (USDA, US) [10], includes over 1,000 *vinifera* accessions (table, wine and unknown type cultivars). The genetic variability of this collection, investigated by the Vitis9kSNP array (9,000 Single Nucleotide Polymorphism), showed a Near East origin of *V. vinifera* and presented evidence of introgression from local *sylvestris* individuals in the cultivated accessions along the European spread routes. The second collection analyzed was the largest grapevines repository located in Vassal (INRA, France) [13], counting for 2,323 unique genotypes representative of the grape growing areas around the world [14]. The microsatellite analysis revealed three main genetic groups and two additional groups, subdividing accessions according to geographic origin (Western regions, Balkans and East Europe, Caucasus and neighbour regions, Iberian Peninsula and Maghreb, Italy and Central Europe) and human use (wine and table grape cultivars).

Allowing the from-East-to-West trend, the genetic variability study of grapevine germplasm (130 grapevine samples representative of *sativa* and *sylvestris* compartments) coming from the first domestication centre, highlighted the uniqueness and originality of Georgian germplasm in respect to the worldwide accessions [12].

Since the ‘80s, different kinds of molecular markers increasingly more accurate, reproducible, repeatable, rapid and less expensive have been developed. The last frontier reached with the new generation sequencing (NGS) technologies is the high throughput SNP genotyping, a whole genome genotyping (WGG) assay that permits the economic and reliable screening of tens/hundreds of thousands markers per assay, leading the molecular characterization using SNP routine. SNP arrays were developed for apple/pear (*Malus pumila* Mill./*Pyrus communis* L.) [15], maize (*Zea mays* L.) [16], peach (*Prunus persica* L.) [17], potato (*Solanum tuberosum* L.) [18] and tomato (*Solanum lycopersicum* L.) [19]. Regarding grapevine, two different high throughput SNP arrays are available, the first one containing 8,898 SNPs [10] and the second one including 18,775 SNPs as part of the GrapeReSeq Consortium [20].

The main objective of this study was to investigate the level of genetic diversity, relationships and structure of dataset obtained by Vitis18kSNP array and to compare the usefulness of this new generation markers system in respect to the traditional SSR (microsatellite) used in [12]. We applied 18 k SNP descriptors, chosen in the frame of GrapeReSeq Consortium, to analyse 71 grapevine accessions representative of wild and cultivated Georgian germplasms, considered valuable genetic resources by the genetic and agronomic point of view.

## Results

### Genetic diversity

**Fig. 1:**
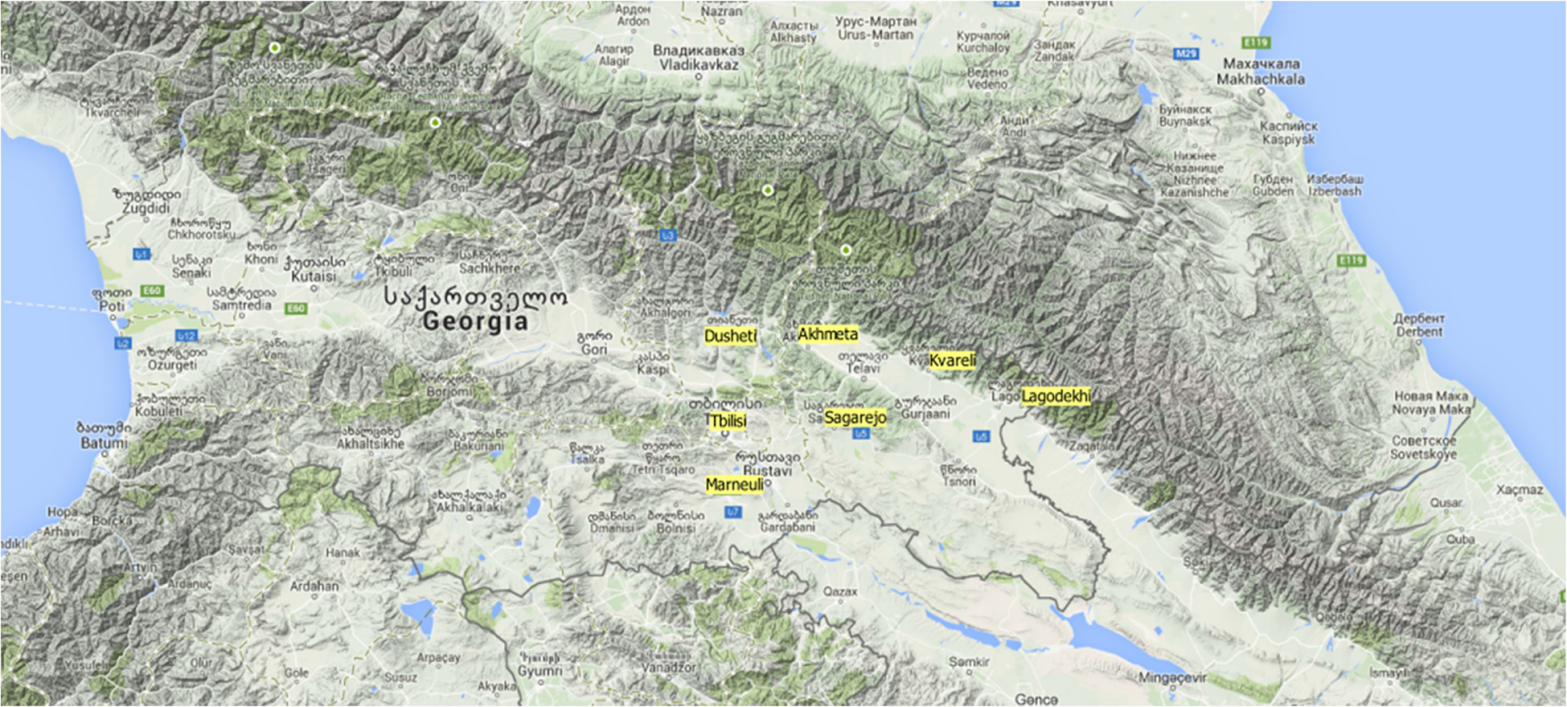
Location of seven Georgian wild populations analysed. The tag of seven wild populations is yellow filled. The image is a Google Physical Layer created in QGIS 2.0

A total of 71 grapevine *sylvestris* and cultivated individuals representative of Georgian germplasm were analysed using the custom Vitis18kSNP array. Information about accession/cultivar name, region of origin, berry colour, flower sex, proles based on Negrul’s observations [21], utilization and localization are given in Table 1 and Fig. 1.

**Table 1:** List of cultivated and wild plant material from Georgia analysed in this work by 18 k SNP loci

ID	Samples	Berry colour^a^	Region of origin	Negrul’s proles	Utilization^b^
	*Vitis vinifera* subsp. *sativa*				
1	Adjaruli Tetri	B	Adjara	*pontica*	W
2	Aladasturi	N	Guria, Imereti	*pontica*	W,T
3	Ananura	N	Kartli	*orientalis*	W
4	Argvetula	N	Imereti	*pontica*	W
5	Asuretuli Shavi	N	Kartli	*orientalis*	W, T
6	Bazaleturi	B	Imereti	*pontica*	W
7	Didshavi	N	Imereti	*orientalis*	W
8	Dziganidzis Shavi	N	Imereti	*pontica*	W
9	Gabekhouri Tsiteli	N	Imereti	*pontica*	W
10	Ghvinis Tsiteli	N	Kakheti	*pontica*	W
11	Gorula	B	Kartli	*orientalis*	T, W
12	Goruli Mtsvane	B	Kartli	*pontica*	W
13	Jani Bakhvis	N	Guria	*pontica*	W
14	Kamuri Shavi	N	Guria	*pontica*	T
15	Khushia Shavi	N	Imereti, Guria	*pontica*	W
16	Kvelouri	N	Imereti	*pontica*	W
17	Marguli Sapere	N	Imereti	*pontica*	W
18	Mgaloblishvili	N	Imereti	*pontica*	W
19	Mrgvali Vardisperi Kurdzeni	RS	Georgia	*orientalis*	T
20	Okhtoura	N	Kakheti	*pontica*	W
21	Orona	N	Guria	*pontica*	W
22	Paneshi	N	Samegrelo	*pontica*	W
23	Rkatsiteli	B	Kakheti	*pontica*	W
24	Rkatsiteli Vardisperi	RS	Kakheti	*pontica*	W
25	Rko Shavi	N	Imereti	*pontica*	W
26	Samarkhi	B	Guria	*pontica*	W
27	Sapena	B	Kakheti	*pontica*	W
28	Saperavi Atenis	N	Kartli	*pontica*	W
29	Saperavi Grdzelmtevana	N	Kakheti	*pontica*	W
30	Shavkapito	N	Kartli	*pontica*	W
31	Tamaris Vazi	N	Kartli	*orientalis*	W
32	Tavkveri	N	Kartli	*orientalis*	W
33	Tchumuta	N	Guria	*pontica*	W, T
34	Tchvitiluri	B	Samegrelo	*pontica*	W
35	Tita Kartlis	B	Kartli	*pontica*	T
36	Tkbili Kurdzeni	N	Kakheti	*pontica*	W
37	Tkvlapa Shavi	N	Imereti	*pontica*	W
38	Tkupkvirta	B	Kakheti	*orientalis*	W
39	Tskobila	N	Kakheti	*pontica*	W
40	Utskveti	B	Racha	*pontica*	W
41	Vertkvitchalis Tetri	B	Imereti	*pontica*	W
42	Zakatalis Tetri	B	Kakheti	*pontica*	W
43	Zerdagi (no true to type)	N	Samegrelo	*pontica*	W
	Samples	Flower sex^c^	Region of origin (district, province)	Site category^d^	Distance from vineyards (km)
	*Vitis vinifera* subsp. *sylvestris*				
44	Bagitchala 05	M	Dusheti, Inner Kartli	A	10.0
45	Baisubani 01	M	Lagodekhi, Kakheti	C	3.0
46	Chachkhriala 01	fruits [F or H]	Akhmeta, Kakheti	AC	10.0
47	Delisi 04	M	Tbilisi, Inner Kartli	C	5.0
48	Delisi 06	M	Tbilisi, Inner Kartli	C	10.0
49	Kvetari 01	M	Akhmeta, Kakheti	C	10.0
50	Kvetari 05	F	Akhmeta, Kakheti	C	10.0
51	Kvetari 10	M	Akhmeta, Kakheti	C	10.0
52	Meneso 02	F	Dusheti, Inner Kartli	C	1.0
53	Misaktsieli 05	F	Dusheti, Inner Kartli	A	1.0
54	Nakhiduri 03	M	Marneuli, Lower Kartli	C	3.0
55	Nakhiduri 05	M	Marneuli, Lower Kartli	C	3.0
56	Nakhiduri 06	M	Marneuli, Lower Kartli	C	3.0
57	Nakhiduri 09	F	Marneuli, Lower Kartli	C	3.0
58	Ninotsminda 04	M	Sagarejo, Kakheti	C	2.0
59	Ninotsminda 08	H	Sagarejo, Kakheti	C	2.0
60	Ninotsminda 09	H	Sagarejo, Kakheti	C	2.0
61	Ninotsminda 11	M	Sagarejo, Kakheti	C	2.0
62	Ninotsminda 13	M	Sagarejo, Kakheti	C	2.0
63	Ramishvili 01	fruits [F or H]	Dighomi collection (Kartli)	-	-
64	Ramishvili 03	fruits [F or H]	Dighomi collection (Kartli)	-	-
65	Ramishvili 05	fruits [F or H]	Dighomi collection (Kartli)	-	-
66	Ramishvili 06	H	Dighomi collection (Kartli)	-	-
67	Ramishvili 07	F	Dighomi collection (Kartli)	-	-
68	Sabue 07	F	Kvareli, Kakheti	C	7.0
69	Sagubari 01	M	Akhmeta, Kakheti	A	6.0
70	Shirikhevi 04	fruits [F or H]	Dusheti, Inner Kartli	A	5.0
71	Zhinvali 01	M	Dusheti, Inner Kartli	AC	10.0

^a^B – Blanc (white), N – Noir (Black), RS –Rose (rose); ^b^W - Wine grape; T - Table grape; ^c^H - Hermaphrodite, F – Female, M – Male; ^d^A - alluvial position (riverbank forest), C - colluvial position (slop of a hill), AC - both alluvial and colluvial positions

The filtered dataset, after the removing of low quality and NC (non-call) loci, counted 15,317 out of 18,775 SNP loci successfully amplified. Among them, 12,083 loci resulted polymorphic, about 79 % of amplified markers. The final SNP allelic profile per each accession is reported in the Additional file 1: Table S1 and is available in Dryad repository [22]. Descriptive statistics for non-redundant genotypes were calculated and the distribution in *sativa* and *sylvestris* groups are summarized in Table 2. In the *sativa* group, were included also some accessions gathered as *sylvestris* but assign to the *sativa* compartment after cluster analysis (see below). The average number of effective alleles was 1.410 and the overall observed and expected heterozygosity values were respectively 0.293 and 0.289, while the percentage of loci showing minor allele frequency (MAF) values > 0.1 was about 73 % and the inbreeding coefficient (F) was 0.011.

**Table 2:** Genetic diversity of Georgian cultivated and wild grapevines revealed by 18 k SNP loci

Compartment/population	N^a^	Ne^b^	Ho^c^	He^d^	MAF^e^	F^g^
*Sativa*	47	1.396	0.312	0.297	24.433	−0.035
*Sylvestris*	21	1.519	0.278	0.329	13.932	0.161
Akhmeta	5	1.307	0.235	0.254	-	−0.087
Dusheti	5	1.326	0.246	0.270	-	−0.098
Kvareli	1	1.911	0.123	0.246	-	-
Lagodekhi	1	1.927	0.124	0.249	-	-
Marneuli	4	1.328	0.247	0.281	-	−0.141
Sagarejo	3	1.294	0.227	0.278	-	−0.203
Tbilisi	2	1.951	0.201	0.257	-	−0.289
Total	68	1.410	0.293	0.289	25.639	0.011

^a^Sample size; ^b^Number of effective alleles; ^c^Observed heterozygosity; ^d^Expected heterozygosity; ^e^Minor allele frequency: percentage of loci having MAF < 0.1; ^g^Inbreeding coefficient; − not detected

The sex ratio (hermaphrodite:female:male) within the *sylvestris* compartment was evaluated (Table 3). The total sex ratio, among the seven populations, was higher for male individuals, followed by female and hermaphrodite (about 62:33:5). While, Sagarejo, Kvareli and Lagodekhi-Tbilisi populations showed the highest percentage of hermaphrodite, female and male flowers, respectively.

**Table 3:** Percentage of male, female and hermaphrodite flowers in seven Georgian wild grapevine populations

Population (district)	Province	Number of individuals	Hermaphrodite (%)^a^	Female (%)	Male (%)
Akhmeta	Kakheti	5	0	40.00	60.00
Dusheti	Inner Kartli	5	0	60.00	40.00
Kvareli	Kakheti	1	0	100.00	0
Lagodekhi	Kakheti	1	0	0	100.00
Marneuli	Lower Kartli	4	0	25.00	75.00
Sagarejo	Kakheti	3	33.30	0	66.70
Tbilisi	Inner Kartli	2	0	0	100.00
Total		21	4.76	33.33	61.90

^a^Accessions classified with hermaphrodite or female flower were scored as female

### Cluster analysis

**Fig. 2:**
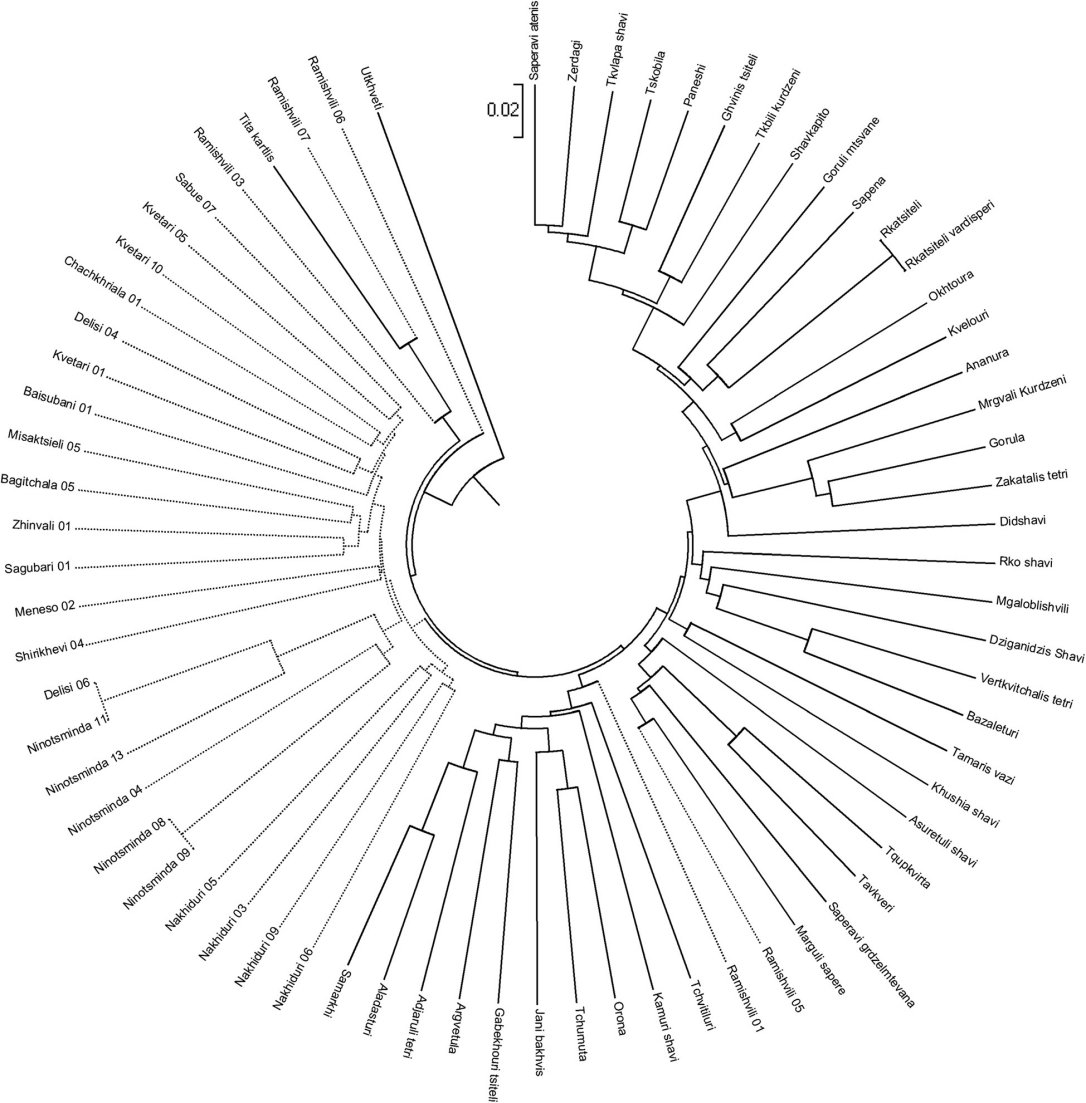
Dendrogram showing relationships among cultivated and wild Georgian genotypes using 18 k SNP loci. Dendrogram generated using UPGMA method. Solid branch lines: cultivated Georgian genotypes; Dotted branch lines: wild Georgian genotypes

The genetic similarity among the different samples was calculated by Dice’s coefficient (PEAS 1.0 software) [23, 24] and the grapevine accessions were grouped in clusters (MEGA 4.0 software) [25] as shown in Fig. 2. The genotypes showed different levels of similarity ranging from 86 and 100 %. Sixty-eight unique profiles were identified, 42 for the *sativa* compartment and 26 for the *sylvestris* compartment. Three pairs of matching genotypes were found, one among cultivars and two among sylvestris individuals.

Using the threshold value of 88 % for similarity index, two main groups were identified, one grouping cultivar samples and one for wild individuals. The 95 % of accessions were clusterized according to accession taxonomic status, except two cultivated genotypes (Tita kartlis and Utskveti, two of the most different genotypes) and two *sylvestris* individuals (Ramishvili 01 and Ramishvili 05) grouped in the *sativa* cluster. In the *sativa* cluster, the cultivars were arranged in two well distinct sub-clusters showing 87 % of similarity and including 18 and 24 unique profiles, respectively. The differentiation among cultivated and wild Georgian compartments was evaluated by Nei’s genetic distance [26, 27] and Fst [28]. The two parameters reached 0.320 and 0.104 values, respectively.

### Population structure analysis and differentiation

**Fig. 3:**
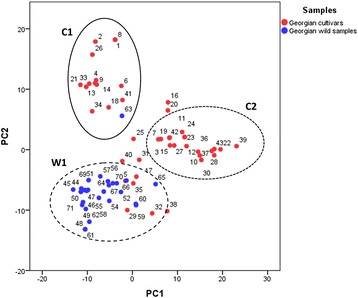
Relationships between wild and cultivated Georgian samples as represented by the first two principal coordinates of PCoA using SNP profiles. C1: Western cultivars; C2: Southern cultivars; W1: wild individuals

In order to identify the structure of populations and the correlations among samples, two different methods were performed. The first method was the PCoA analysis [29], computed based on the genetic distance matrix obtained by SNP profiles. Two dimensional projections of PCoA analysis per each sample were plotted in a 2-D dimension scattered plot (Fig. 3). The first two principal components (PCs), accounting for 25.63 and 18.29 % of the total variation, differentiated clearly the genotypes into 3 main groups, despite the presence of overlapping areas: two groups including cultivars (C1 and C2) and one for wild individuals (W1). In the overlapping areas, several cultivated samples appeared borderline with W1 samples. Along the PC1, a separation between C2 and W1 groups was highlighted, while the discrimination of C1 group was highlighted by the PC2.

**Fig. 4:**
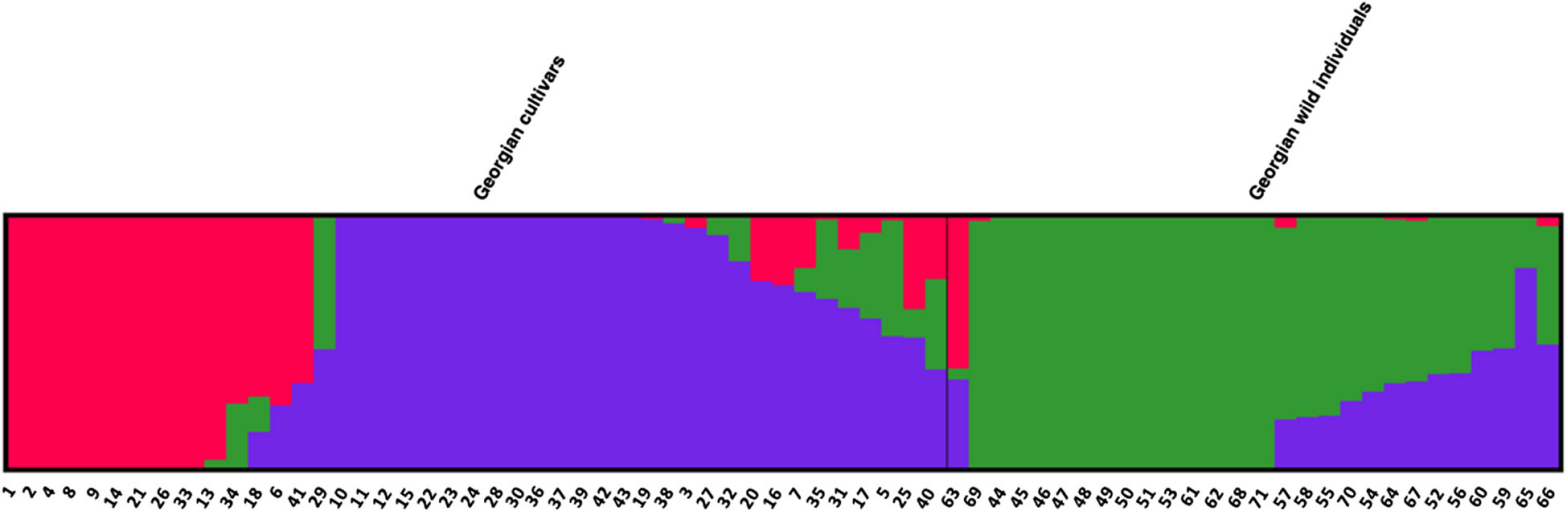
Admixture proportions of wild and cultivated Georgian groups, as estimated by fastSTRUCTURE at K = 3, displayed in a barplot. Each sample is represented as a vertical bar, reflecting assignment probabilities to each of the three groups. G1: red bars; G2: purple bars; G3: green bars

The second method used to infer the relationship among genotypes was the clustering algorithm implemented in the fastSTRUCTURE program [30]. In order to uncover the hierarchical population structure, different numbers of K populations were explored (Fig. 4). Optimal K estimated the most likely number of populations at K = 3. Using a >0.75 % threshold for group assignation, 48 samples (68 %) were assigned to a cluster at K = 3 (Additional file 2: Table S2). Structure clustering highlighted 3 groups: two groups for *sativa* samples (G1 and G2) and one for *sylvestris* individuals (G3), including 25, 42 and 33 % of the entire genetic pool, respectively. In G3, only putative wild accessions (89 %) were included. The inbreeding coefficient (Fst) within three subpopulations identified by STRUCTURE analysis ranged from 0.076 (G1-G2 pairwise) to 0.064 (G2-G3).

### Parentage analysis

Pairwise IBD (identical-by-descent) analysis was used to investigate the first-degree (PO: parent-offspring) and second-degree relationships among the wild and cultivated Georgian individuals by PLINK [31]. For an ideal situation without genotyping errors and/or mutations, Z0 (probability to share 0 IBD alleles) and Z2 (probability to share 2 IBD alleles) of PO pairs are expected to be 0 and Z1 (probability to share 1 IBD allele); Z0 and Z1 of 2^nd^ degree pairs are expected to be 0.5 and Z2 to be 0. Therefore, pairs of genotypes holding a PI-HAT (relatedness measure) value similar to 0.5 are related by first-degree or closer relationships. Two pairs of individuals (Table 4) having Z0 and Z2 near 0, Z1 values higher than 0.9 and with relatively high proportion of IBD (PI-HAT ≈ 0.5) were considered PO pairs. One PO pair was identified between two wild samples (Ninotsminda 11 - Ninotsminda 13) and one between wild and cultivated samples (Ramishvili 07 - Tita kartlis). While, five pairs of samples (Table 4) with proportion of IBD (PI-HAT) ≈ 0.25 and relatively high Z0 and Z1 (≈0.5) values were considered 2^nd^ degree pairs. The remaining pairs of individuals were considered “unrelated” according to the relationships identified. No 2^nd^ degree relationships were identified among wild accessions and wild and cultivated samples.

**Table 4:** Parentage analysis and relationship categories assignment (RCA) for wild and cultivated Georgian grapevines obtained by SNP allelic profiles

Sample 1	Sample 2	Z0^a^	Z1^b^	Z2^c^	PI-HAT^d^
*RCA: Parent-Offspring*					
*Ninotsminda 11*	*Ninotsminda 13*	0.0174	0.9015	0.0811	0.5318
*Ramishvili 07*	Tita Kartlis	0.0000	1.0000	0.0000	0.5000
*RCA: 2* ^*nd*^ *degree*					
Ghvinis Tsiteli	Tkvlapa Shavi	0.4841	0.4889	0.0397	0.2842
Mrgvali Kurdzeni	Zakatalis Tetri	0.4606	0.5076	0.0068	0.2606
Paneshi	Saperavi Atenis	0.5552	0.4709	0.0698	0.3053
Saperavi Atenis	Shavkapito	0.4807	0.5288	0.0163	0.2807
Saperavi Atenis	Tkbili Kurdzeni	0.4693	0.5103	0.0142	0.2694

^a^probability to share 0 IBD allele; ^b^probability to share 1 IBD allele; ^c^probability to share 2 IBD allele; ^d^relatedness measure. *Italic type*: putative *V. vinifera* subsp. *sylvestris* individual

## Discussion

### Genetic variability of Georgian *sativa* and *sylvestris* germplasms

In order to develop appropriate strategy for long-term conservation of the Georgian (and more general Caucasian) grapevine biodiversity, the identification and characterization of genetic resources is mandatory. There are not definitive data giving an estimation of the number of autochthonous varieties in this area: 525 varieties are listed in the Ampelography of Georgia [32], only 414 were described in the Ampelography of the Soviet Union (1947–1970), but only 248 remained in old collections until 2003 [33]. In the present study, the new Vitis18kSNP array, containing 18,775 SNP markers, were used to analyse the genetic relationship among a dataset of cultivated (43) and putative wild (28) grapevine accessions belonging to the autochthonous germplasm of Georgia.

The SNP statistic parameters calculated to determine the genetic diversity of Georgian germplasm reflected the results published in [12], regarding the genetic variability investigated by SSR markers. Considering the difference in the number of analysed accessions and the kind of molecular markers, the trend of Ne (number of effective alleles), Ho (observed heterozygosity) and He (expected heterozygosity) values between *sativa* and *sylvestris* compartments were almost comparable with the values evidenced in the previously cited work and in other works devoted to the study of cultivated and wild grapevines [11, 34]. For *sativa* compartment, the Ho value appeared slightly higher than the He value; while for wild accessions, the trend was opposite. The Ho reduction observed overall *sylvestris* samples and among populations was detected also by other studies [8, 34-39]. It indicated that the wild individuals suffer from inbreeding. This result was not observed for wild grapevine populations of Tunisia [40], as well as for the 18 spontaneous growing vines from Georgia analysed in [12]. The MAF value was higher for cultivated than wild samples, while, F showed mean value higher for *sylvestris* individuals (overall samples and among populations) than cultivars, and the same trend reported in [34] was displayed. MAF and F values were consistent with Ho results, showing that *sylvestris* compartment is more inbreed than the *sativa* compartment.

One of the main morphological distinctive traits between wild and cultivated grapevine forms is the flower sex, mostly hermaphrodite for cultivars and male or female for wild grapevine [4]. Moreover, hermaphrodite wild grapevine plants were also gathered. Subspecies *sativa* is self-pollinating, while subsp. *sylvestris* has an anemophilous and entomophilous pollination [41]. In nature, it was found a predominance of male wild grapevine individuals [42, 43]. Our results fit this evidence. Because of the flower of wild grapevines is unisexual and pollen of male plant fertilizes the ovary of female plant, the reproduction *via* sexual pathway of Kvareli, Lagodekhi and Tbilisi populations, where only female or male plants were collected, resulted damaged and these population are seriously endangered. Based on recent surveys in various European Countries [44-47], the wild grapevine populations appeared severely endangered and the reasons could be addressed to the human activities, ecosystem fragmentation events and spreading of Northern American pathogens. Nevertheless, in the natural environment, Georgian wild grapevine individuals did not show any signs of phylloxera attack. This could be explained because the existence of disease symptoms in wild individuals was verified only when the pest is directly and artificially inoculated [47].

Moreover, due to the limited number of individual per population our conclusions about their fitness are not really robust and have to be considered preliminary. Further surveys, devoted to explore in detail the spontaneous grapevine populations in Georgia and Caucasus as well, were conducted in the frame of EU project COST Action FA1003 “East–west Collaboration for Grapevine Diversity Exploration and Mobilization of Adaptive Traits for Breeding”. Fourteen wild populations were investigated in their natural environmental (more than 100 individuals were sampled) and a prospecting on the sanitary status of the aerial organs and roots was carried out (Maghradze et al. accepted in Vitis). A genetic analysis including individuals coming from the latter surveys could give more exhaustive information regarding genetic diversity, fitness and inbreeding rates of grapevine wild populations in the Caucasus region.

In both *sativa* and *sylvestris* compartments, samples sharing the same allelic profile were found, for a total of 68 unique profiles identified (Fig. 2). Among the cultivars, the two samples sharing the same allelic profiles were Rkatsiteli and his berry colour mutant Rkatsiteli Vardisperi [12].

Rkatsiteli Vardisperi, a pink-wine grape, is a Rkatsiteli clone selected by V. Loladze in 1948 [48]. *V. vinifera* subsp. *sativa* is a cultigen vegetatively propagated through cuttings or budding. During this reproductive pathway, mutagenic events in the somatic cells of buds could take place and if they are used for propagation they lead to genotype having phenotypic traits different to the mother grapevine. In the *sylvestris* compartment, two Ninotsminda individuals (08 and 09) collected in the same area, Sagarejo, shared the same allelic profile, while another accession (Ninotsminda 11) showed the same SNP profile of Delisi 06, an accession coming from Tbilisi, about 60 km far from Sagarejo (Fig. 2). The identification of two identical accessions (Ninotsminda individuals) collected in the same area could be addressed to a vegetative propagation event occurred to ensure a rapid vine regeneration and soil colonization. On the other hand, an error sampling could be highlighted for Ninotsminda 11 and Delisi 06.

In order to determine the genetic relatedness among genotypes, a clustering analysis was carried out (Fig. 2) and the results were validated by pairwise Nei’s genetic distance and Fst values. A clear differentiation regarding *sylvestris* and *sativa* compartments was recognized, using a threshold value for the similarity index lower than 87 %. Moreover, the result represented in Fig. 2 clearly showed that genetic distances are directly proportional to regional distances: the *sativa* samples were arranged based on the Western and Eastern origin, while the most part of *sylvestris* individuals were grouped according to their region of origin [12], *e.g.* Kvetari’s, Nakhiduri’s and Ninotsminda’s.

The Utskveti variety, a cultivar clustering very distinct from the other ones, was interesting, as well as Tika kartkis variety, grouped together with Ramishvili wild individuals. The Utskveti variety was originated and widely spread in the past years in Racha province [49], but recently is only maintained in collections. The name of this variety was mentioned in the list of Georgian local varieties [32] and the ampelographic description has been available since 1939 [50]. It is a white berry wine grape variety with strong hairs on lower leaf surface and with very dense bunches. The phenotypical observation of Utskveti accessions in the available Kindzmarauli, Telavi and Saguramo collections were only partially in agreement with the bibliography. Nowadays, the accessions have white berry and dense bunches but hairless lower leaf surface. Thus, some doubts about the correspondence of these accessions with historical Utskveti grape have to be accounted.

In the grapevine germplasm collections of Georgia are preserved two genotypes called Tita Kartlis. One is the true-to-type Georgian cultivar Tita Kartlis, having deeply lobed leaf and small prolonged berries [42] and the other genotype is the Azerbaijani cultivar Tabrizi, known in Georgia with synonym name of Ganjuri, differing from the Tita Kartlis true-to-type because of less lobed leaves, prolonged but larger berries and teeth in the petiole sinus [32]. Since the ampelographic description of the analysed accession in this study corresponds to the description reported in Ampelography of Georgia [32], the identification of Tita Kartlis is not questionable.

Taking into account that the Southern Caucasus (Armenia, Azerbaijan and Georgia) has been considered the first centre of grapevine domestication [7], the existence of local cultivars presenting morphological and genetic traits similar to wild individuals could be an instance of hybridization and introgression events among wild and domesticated accessions. Those events due to pollen flow between cultivars and wild forms were previously proved [11, 51] and could have severe consequences in the conservation of wild grapevine populations and advance the doubt if the current wild populations fit the ancestral grapevine forms [51]. Moreover, there are signs that only few Georgian cultivars could correspond to stocks introduced in the past from other neighbouring regions or far away countries, as France [12]. Despite the clear distinction between *sativa* and *sylvestris* compartments, few wild samples clusterized together with the cultivated samples. It is the case of Ramishvili samples, two grouped in the *sativa* cluster and three in the group of samples clusterized as outgroup. The Ramishvili samples have been collected by professor Revaz Ramishvili during his survey around Georgia in order to collect and study wildly growing grapevines. During this survey, not only wild grapes *V. vinifera* subsp. *sylvestris* were collected, but also accessions discovered in wild conditions during his expeditions and showing a phenotype holding typical ampelographic traits (grapes and leaves) of both *sylvestris* and *sativa* subspecies [52]. Based on cluster analysis, Ramishvili 01 and Ramishvili 05 could be considered cultivars because of their grouping in the dendrogram (Fig. 2). Regarding the accession Ramishvili 03, we do not have information about the flower sex, but we know it has white berries and we could conclude that it is not likely a *V. vinifera* subsp. *sylvestris* [53]. The accession Ramishvili 06 is hermaphrodite, whereby we could exclude its wild nature and classify it in the domestic compartment, as well as the accession called Ramishvili 07, having a female flower but not a wild habitus.

The identification of two well distinct clusters for Georgian samples were consistent with the high genetic variability and the genetic diversity of Caucasus germplasm coming from Georgia, considered a primary centre of grapevine domestication [7, 12, 13]. The high polymorphism of Georgian grapevines was also discovered by morphological characterization of *sylvestris *populations [54].

The two main groups obtained by cluster analysis were confirmed by Nei’s genetic distance value (0.320), that it reflected the 87 % of similarity between the *sativa* and *sylvestris* clusters. This evidence was in agreement with the gene flow between the wild and cultivated compartments [11, 12]. On the other hand, the Fst value, accounting 0.104, meant that the two groups have a moderate differentiation based on the interpretation suggested by Wright [28]. This interpretation did not fit the low level of genetic differentiation between Georgian wild and cultivated grapevines revealed by using a moderate number of microsatellite loci [12, 55] or between Eastern *sativa* and *sylvestris* accessions analysed by 9 k SNP loci [10]. The latter discrepancy could be due to the absence of Georgian cultivars and the restricted number of Georgian wild individuals in the dataset.

Significant Fst values of genetic differentiation (about 0.140) have been reported between grapevine accessions of *sylvestris* and *sativa* in Morocco [38] and in Spain [11].

In agreement with the cluster analysis, the PCoA performed to identify the potential correlations among populations, revealed three main groups: C1, C2 and W1 (Fig. 3). Similar results, a clear distinction between *sativa* and *sylvestris* compartments, were also found analysing the Northern African germplasm by 20 nuclear microsatellites [40]. A differentiation of two separate clusters among Georgian cultivated samples was showed, confirming the existence of two genetic groups within the Georgian *sativa* germplasm, following the geographical provenience in the Georgian country described in [12] and [52], based on the molecular and morphological characterization, respectively. The samples collected in the Eastern regions of Georgia appeared separate from the accessions collected in the Southern and Western regions due to the orography and river basins functioned as biological boundaries. The overlapping area between C2 and W1 groups, slightly flattening the differentiation of cultivated and wild germplasm, was consistent with Nei’s genetic distance value obtained between *sativa* and *sylvestris* compartments and the discrete degree of similarity between the *sativa* and *sylvestris* subspecies [34], pointing out the existence of gene flow between both compartments [11, 12, 53]. Based on this evidence, it could be advanced the hypothesis of existing intermediate genotypes, having ampelographic characteristics inherited by both *sativa* and *sylvestris* subspecies, due to potential domestication events occurred in the past years in this area. Indeed, Ramishvili accessions could support this hypothesis: Ramishvili 05 was placed in between the C2 and W1 groups and Ramishvili 03, 06 and 07 accessions, considered *sativa* samples based on cluster analysis, in the PCoA plot belonged to W1. As well as, the clustering of six cultivars (Asuretuli Shavi, Marguli Sapere, Saperavi Grdzelmtevana, Tita Kartlis, Tavkveri and Tkupkvirta) in the W1 group led us to suppose that these cultivars were derived from local domestication events of *sylvestris* individuals. Contrary to what has been observed in this work, Asuretuli Shavi, a black berried female variety from the Southern Georgia (Marneuli district), was identified as a case of doubtful Georgian origin, because of based on SSR genotyping it showed a PO relationship with the ancient Greek variety Rhoditis [12]. Likewise the cluster analysis, Ramishvili 01 accession was grouped in one of the two *sativa* groups (C1). While Utskveti, the cultivars showing the highest genetic diversity in respect to the entire set of samples, was placed in the overlapping zone between C2 and W1. Furthermore, the distance between *sylvestris* sites and vineyards appeared to do not influence the overlapping area.

In addition to the major partition in cultivated and wild groups, STRUCTURE analysis identified three significant genetic groups, G1, including the majority of cultivars coming from Western region, G2, clustering *sativa* samples with predominance of cultivars coming from Eastern Georgia and G3, the group consistent with the wild accessions (Fig. 4). The STRUCTURE results, with 68 % of accessions clearly assigned to one group, recognized the genetic structure of Georgian germplasm (*sativa* and *sylvestris*), while the existence of samples showing an unclear assignation (less than 75 % of probability, Additional file 2: Table S2) could reflect the events of genetic introgression between wine-growing areas of Georgia. Considering the putative wild individuals analysed in this study, 14 out of 28 samples showed a percentage of assignation higher than 95 %, leading us to hypothesize that these wild individuals could be considered ancestral grapevine forms. Indeed, the accessions belonging to Ramishvili group were mostly included in G1 and G2 (Ramishvili 01, 05 and 06) and the other ones showed about 34 % of assignation to the Eastern Georgia group. The same six cultivars grouped into the W1 of PCoA plot were included in G2 and showed a not negligible percentage of assignation to G3. The pairwise Fst values higher than 0.05 among G1, G2 and G3 subpopulations revealed a moderate differentiation and the relatedness between Eastern and *sylvestris* individuals groups was confirmed by Fst lower value for G2-G3 pairwise. These results suggested that domestication events occurred in this geographic area as well as identified in [54, 55], where the STRUCTURE analysis, carried out on Georgian and wild accessions, revealed admixture among cultivated and wild samples, but a clustering regardless of their collection region was observed.

Archaeological evidence suggests that the grapevine domestication took place in South Caucasus and that its spread followed successive scenarios: the first one from Caucasus toward South-West (Eastern Mediterranean Countries), the second one toward Anatolia and after on the way to Greece, Balkans, Sicily, Southern Italy, France and Spain and the last one from France to Central Europe [7, 56]. Moreover, secondary centres of domestication have been proposed, as well as Iberian Peninsula, where it was found the chlorotypes of *sylvestris* and *sativa* genotypes compatible with Western cultivars chlorotypes [9], and Italy, where the allelic profile of some cultivars was found very similar to some wild accessions [57].

Even though a connection between some *sylvestris* and *sativa* individuals was highlighted by both multivariate and STRUCTURE analysis, the kingship analysis did not find out close relationship between wild and cultivated samples, because of Ramishvili 07, showing a PO relationship with Tita Kartlis, is now considered a *sativa* individual. Nevertheless, if introgression events occurred between the two subspecies and parental individuals were not analysed, the parentage relationships higher than 2^nd^ degree are difficult to identify. Moreover, it cannot be excluded that close relationship could be discovered between two subspecies enlarging the number of analysed accessions. The 1^st^ degree relationship between two wild samples (Ninotsminda 11 and Ninotsminda 13), located in sites not far from each other is consistent with propagation events by seed dispersal [58] and confirmed the inbreeding tendency in some wild populations.

In a time characterized by great challenges to face climatic change and to develop sustainable agricultural models based on use of moderate irrigation, fertilisation and pesticides, the selection of new genotypes for ensuring an optimal productivity in terms of quality and quantity is mandatory. It was demonstrated that the Georgian grapes are late ripening cultivars, characterized by a long vegetative and reproductive development (from bud break to harvesting time) in comparison with Western European cultivars [59]. The objective to select varieties showing a wider range of phenological variability and genetic traits, apparently not represented in the germplasm of Western Europe, makes the Georgian varieties a considerable background for grapevine breeding programs aimed to extend the ripening time in a viticultural area and consequently reducing possible berry summer stresses and grapes quality impairment.

Considering the grapevine defence against diseases, a survey about use of fungicides in member states of the European Union highlighted that viticulture accounts for approximately 70 % of all agrochemicals used. Nevertheless, an intensive use of chemicals becomes more and more unsustainable because of high costs, and possible negative impact on environment and human health due to the chemical residues in grapes, soil and aquifers. The EU Directive 2009/128 for sustainable control of diseases caused by plant pathogens in Europe strongly recommends a decrease in the number of pesticide treatments carried out in the field. Thus, following the first interesting results obtained by screening the Caucasian germplasm [60], a systematic investigation of Georgian grapevine genetic resources, searching for resistant traits to pathogen, seems to be a promising strategy for plant breeding programs aimed to reduce the fungicides use in vineyard assuring at the same time an acceptable protection against pathogens.

### SNP and SSR molecular markers in comparison

Vitis18kSNP array is the largest SNPs set implemented in a high-throughput genotyping technology for genetic diversity in grapevine. The previously SNPs sets included tens [61], hundreds [34] or thousands loci [10]. SNP platforms have been developed following the huge genomic data obtained by sequencing and re-sequencing of whole genomes using NGS technology on accelerated pace, which allow high-throughput and low cost genotyping of thousands of markers in parallel.

On the other side, SSR markers are a useful instrument widely used for genotyping, to solve problems of homonymies, synonymies and kinships, to infer genetic structure of populations in wild and cultivated grapevines [11-14, 32, 62, 63]. A set of 9 SSR markers was proposed as minimal set of loci for genotyping routine analysis [14] and for parentage analysis or for germplasms not covered by this set an additional group of 13 SSR loci was included [14, 63].

In this work, it was demonstrated that the SNP markers were useful for germplasm management, as already observed in grapevine [10] and in many other species [15, 17, 18] and that the results could be compared to other marker systems, as the traditional SSR [12, 55]. Moreover, SNP markers revealed a higher differentiation, pointing out a moderate differentiation between *sativa* and *sylvestris* compartments based on Fst value, and at the population level the high number of loci should solve better the genetic relationship among samples. In respect to SSR markers, these microarray-based markers were used to investigate helpfully the genetic diversity of Georgian *sativa* and *sylvestris* germplasms with a limited expense in terms of time and money and obtaining a high data reliability (only the 18 % of loci showed low quality or were not detected). Moreover, since the SNPs are biallelic the genetic profiles could be easily compared to datasets generated by other laboratories around the world, without incurring problems related to difficulty on data standardization [64].

Another winning aspect could be the application of Vitis18kSNP array for parentage analysis. Nowadays, the parentage analysis works are carried out including dozens of SSR loci [63] and, sometimes, even by increasing the number of analysed loci not all the relationships discovered previously can be ruled in [65]. An in-deeper analysis, using thousands of SNP loci, could strengthen the data obtained by kinship analysis, mostly for second and third-degree relatives, for which more than 50 SSR loci should be investigated for the detection [66].

Furthermore, this array could successfully be chosen for the construction of high-density maps, quantitative trait loci (QTL) mapping, genetic diversity and parentage analysis in grapevine.

## Conclusions

The results obtained by molecular analysis of Georgian germplasm using a large set of SNP markers provided information of high genetic diversity of *sativa* and *sylvestris* Georgian germplasms, as previously investigated by other molecular markers and by morphological evaluations. Our data showed that the Vitis18kSNP assay can be used successfully for high-throughput SNP genotyping in grapevine and represented a viable alternative to traditional genotyping techniques. According to this work, a moderate differentiation between *sativa* and *sylvestris* compartments was discovered, due to centuries long separation of two taxa, making it quite impossible to trace the events of *V. vinifera* domestication. On the other hand, connection between samples of both subspecies may be assumed as well, highlighting the occurrence of cross hybridization events among native wild populations and cultivars.

## Methods

### Plant materials and DNA extraction

In this study, 43 cultivated samples and 28 putative wild accessions coming from Georgia and maintained in the germplasm collections of University of Milano were considered. A detailed list of plant material is reported in Table 1. About *sylvestris* accession sampling, refer to Material and Methods described in [12]. Seven grapevine wild populations were taken into account in this work, distinguished on the basis of some parameters, such as sharing of the same area, distance between groups (more than one linear kilometer) or the presence of geographical barriers (Fig. 1). Ramishvili samples were covered as *sylvestris* individuals from Dighomi collection (Kartli, Georgia). Accessions were classified in the *V. vinifera* subsp. *sylvestris* taxon according to their expected morphological traits, mainly related to the young and mature shoots and leaves, flower type and bunch aspect at flowering and during ripening, berry and seed size and shape. This morphological analysis allowed also to discriminate among true *V. vinifera* and possible non *V. vinifera* species or inter-specific hybrids. In particular accessions were considered genuine wild *V. vinifera* if they showed: i) fully opened young shoot apex; ii) low anthocyanin coloration and density of hairs, both on young shoot apexes and leaflets; iii) mature leaves small or medium in size, with short teeth, low density of hairs and open petiole sinus; iv) small bunches; v) small and round berries; vi) roundish pips.

Total genomic DNA was extracted by young leaves using the DNeasy™ Plant Mini Kit (Qiagen - Hilden, Germany). In order to determine the DNA quality, the 260/230 and 260/280 ratios was detected by NanoDrop Spectrophotometer (Thermo Scientific - Waltham, Massachusetts). Quant-iT PicoGreen Assay (Invitrogen - Carlsbad, California) was used to quantified the DNA concentration.

### SNP genotyping

The 18,775 SNPs contained in the Vitis18kSNP array (Illumina Inc., San Diego, California) were analysed. Two hundred nanograms of genomic DNA were delivered to Fondazione Edmund Much (San Michele all’Adige, Trento, Italy) and were used as template for the reaction, following the manufacturer’s instructions (Illumina Inc.). Nucleotides were scored with Genotyping Module 1.9.4 of the GenomeStudio Data Analysis V2011.1 software (Illumina Inc.). Dataset was filtered based on SNP call quality and GenTrain score: samples with low SNP call quality (*p50GC* < 0.54) were removed from the analysis and only SNPs with a GenTrain score higher than 0.6 were retained. Markers with a number of NCs (non-call) higher than 20 %, as well as the 100 % NC markers, were removed. The data can be downloaded from Dryad repository (De Lorenzis et al. [22], http://dx.doi.org/10.5061/dryad.521h5).

### Data analysis

In order to estimate the genetic diversity of Georgian germplasm, the SNP genotyping data were used to determine the effective number of alleles (Ne), the observed heterozygosity (Ho), expected heterozygosity (He) [67], the minor allele frequency (MAF) and inbreeding coefficient (F), performed by PEAS 1.0 software [23]. The sex ratio of *sylvestris* individuals was calculated among and within populations, estimating the percentage of hermaphrodite, female and male flowers.

MEGA software (version 4.0) [25] was used to design a phylogenetic tree by the UPGMA (Unweighted Pair Group Method with Arithmetic Mean) method. The SNP distance matrix was generated by PEAS 1.0 software [23] based on the Dice’s coefficient [24]. The validation of clustering results was performed considering the pairwise Nei’s genetic distance [26, 27] and pairwise Fst analysis [28]. The parameters were carried out using the pp.fst function of HierFstat package [68] and nei.dist function [69] of R program.

The structure and the association between *sativa* and *sylvestris* Georgian compartments were investigated following two different approaches: i) Principal Coordinates Analysis (PCoA) [29], used to capture the correlation between genotypes; ii) STRUCTURE analysis [30], a Bayesian approach attempts to interpret the correlation between genotypes in terms of admixture between a defined number of ancestral populations. The PCoA analysis was carried out by GenAlEx 6.501 software [69], starting the correlation matrix. The STRUCTURE analysis was carried out using fastSTRUCTURE software package [30], using the input files (.bed, .bim, .fam) generated by PLINK 1.07 software [31]. K (number of ancestral genetic groups) values, ranging from 1 to 10, were tested by 10 iterations per each K and the most likely K value was chosen, running the algorithm for multiple choices of K. The admixture proportions estimated the most likely K was viewing by DISTRUCT software [70]. The K clusters obtained by STRCUTURE analysis were validated performing pairwise Fst values [28].

In order to infer relationships among individuals, we employed the PLINK 1.07 software [31] on each pair of all the genotypes (only unique genotypes were included), estimating the proportion of the SNPs at which there were 0, 1, and 2 shared alleles identical-by-descent (IBD: probability of two genotypes are descended from a single ancestral genotype and not identical by chance), denoted by Z0, Z1, and Z2 respectively and PI-HAT values, the relatedness measure measured as PI-HAT = P (IBD = 2) + 0.5 x P (IBD = 1). The parameters, minor allele frequency (MAF) and *r*^*2*^ of linkage disequilibrium, were set on 0.01 and 0.05 values.

### Availability of supporting data

The data set supporting the results of this article is available in the Dryad repository, (De Lorenzis et al. [22], http://dx.doi.org/10.5061/dryad.521h5) and as complementary material (Additional file:1: Table S1).

## Additional files

Additional file 1: Table S1. SNP allelic profile of 43 grapevine cultivars and 28 putative wild individuals from Georgia at 15,317 SNP loci. Dataset resulted was filtered based on SNP call. Additional file 2: Table S2. Ancestry values (mean and standard deviation values over 10 interations) for the three genetic groups inferred by structure on 43 grapevine cultivars and 28 putative wild individuals from Georgia genotyped at 15,317 SNP loci.

## Abbreviations

A: Alluvial position (riverbank forest)
AC: Both alluvial and colluvial positions
B: Blanc (white)
C: Colluvial position (slop of a hill)
F: Female
F: Inbreeding coefficient
Fst: Fixation index
H: Hermaphrodite
He: Expected Heterozygosity
Ho: Observed Heterozygosity
IBD: Identical-by-Descent
M: Male
MAF: Minor Allele Frequency
N: Noir (Black)
N: Sample size
NC: Non-Call
Ne: Number of effective alleles
NGS: New Generation Sequencing
PC: Principal Coordinate
PCoA: Principal Coordinate Analysis
PI-HAT: Relatedness measure
PO: Parent-Offspring
QTL: Quantitative Trait Locus
RS: Rose (rose)
RCA: Relationship Categories Assignment
SNP: Single Nucleotide Polymorphism
SSR: Simple Sequence Repeat
T: Table grape
W: Wine grape
WGG: Whole Genome Genotyping
Z0: Probability to share 0 IBD allele
Z1: Probability to share 1 IBD allele
Z2: Probability to share 2 IBD alleles
